# Polish Experiences of Safety Measures Involving Forensic Psychiatric Inpatients Implemented During the SARS-CoV-2 Pandemic

**DOI:** 10.3389/fpsyt.2020.576703

**Published:** 2021-01-15

**Authors:** Janusz Heitzman, Paweł Gosek

**Affiliations:** Department of Forensic Psychiatry, Institute of Psychiatry and Neurology, Warsaw, Poland

**Keywords:** COVID-19, mental health care, forensic psychiatry, safety, pandemic

## Abstract

The SARS-CoV-2 pandemic has made it necessary for us to adapt our healthcare systems to a very different sort of reality. This clearly also applies to psychiatric services. The restrictions and safeguards associated with the pandemic particularly concern adherence to social distancing and medical treatment safety procedures. The implementation of these procedures is generally complicated by conditions of forensic psychiatry where, in line with demands made by courts, the treatment and isolation of mentally unwell offenders must be carefully managed. In most countries, forensic psychiatric treatment is an inpatient service where patients are kept in restricted and cramped spaces, making social distancing difficult to implement as patients participate in compulsory group therapeutic activities. As a result, it is necessary to introduce unique recommendations relating to patient safety and treatment adapted to the realities of forensic psychiatry. All this requires the implementation of additional restrictions, over and above those arising from the essential aspects of forensic psychiatry. In this paper, we present and discuss the Polish guidelines for forensic psychiatric care during the SARS-CoV-2 pandemic, developed as a result of discussions on essential measures introduced to reduce the spread of the virus and the unique needs of the forensic patient population.

## Introduction

The SARS-CoV-2 virus pandemic makes it necessary for us to adapt our healthcare systems to meet the needs of this new reality. This also applies to psychiatric services, both general and forensic psychiatric types. The restrictions associated with the pandemic concern, in particular, the maintaining of social distancing and safety during medical procedures. In many countries, restrictions have reached the level of government laws and regulations, with financial penalties and even prison terms being imposed upon those who breach restrictions. The international community of scientists and medical practitioners has developed a number of guidelines on how to reduce the risk of SARS-CoV-2 virus infection – as hospitals are environments where the risk of infection is relatively high, compliance with safety procedures is particularly important there. The implementation of these guidelines is generally hindered by the conditions of forensic psychiatry, where, in line with demands made by the courts, the treatment and isolation of mentally unwell offenders is provided. In most countries, forensic psychiatric treatment is an inpatient service where patients are forced to permanently stay in small, restricted spaces, most often having meals in a common room and participating in compulsory therapeutic group activities. As a substantial number of forensic inpatients suffers from various physical conditions and comorbidities, for example relating to substance misuse, such as malnutrition and impaired functioning of the immune system, they are much more vulnerable to Coronavirus disease 2019 infection (SARS-CoV-2, COVID-19) than the general population (1). In the Polish legal system, decisions relating to the location of forensic psychiatric treatment are made only by competent courts (2). Therefore, in cases of emergency evacuation of patients due to sanitary emergencies, this is a particularly difficult procedure. As seen in the example of the 1918 Flu Pandemic, viral transmissions can be much more advanced among isolated individuals, such as those detained in prisons (3). Moreover, any additional stressor acting in particularly susceptible people, such as forensic psychiatric patients, may cause trauma and result in the intensification of mental disorders in the future, including, among others, increased anxiety (4). Another issue, no less important, is the restriction of civil rights for patients detained in court-imposed psychiatric centers. Restrictions resulting from the SARS-CoV-2 pandemic affect the general public, but must be implemented with special care for persons deprived of their liberty. Such a situation makes it necessary for the medical establishment to introduce specific recommendations concerning the safety of treatment and stay of patients adapted to the realities of forensic psychiatry. In recent months, several authors have written to suggest an intensified level of care be provided for detained and imprisoned psychiatric patients. This concerns, among other things, the continuity of treatment, coordination of treatment and care between different institutions, psycho-education, the provision of personal protective equipment and appropriate safety procedures, and the filling of staff shortages relating to those working in general psychiatry on a daily basis (5, 6). Kennedy et al. have noted that the Covid-19 pandemic has accelerated the introduction of tele-medicine technical solutions as telepresence in courts, and changed the hospital daily routine involving, among others, the introduction of disinfection, physical distancing, screening staff, ending visitor access and leave for patients, organizing isolation wards, quarantining of admissions, etc. (7). Italian clinicians and researchers from the Lombardy province have stressed that practitioners should be equipped with appropriate e-health technologies and procedures to cope with epidemic conditions. The videolink or other tele-medicine services should also be accessible to caregivers and families. Another issue mentioned is the development of interventions which mitigate the potentially harmful consequences of quarantine, as in the example of self-help groups (8).

In this article, we present and discuss the safety procedures and recommendations related to SARS-CoV-2 virus pandemic, based on clinical experiences in the Polish forensic psychiatry environment, to share the Polish solutions and experiences in this area. Mentioned recommendations are one of the first to be published in the field of forensic psychiatry and can make an important contribution to the discussion on the shape of care in individual countries. The discussion and comparison of solutions in different countries will also allow to work out the best operational procedures, which can be extremely useful during possible similar threats in the future. In addition to the threats and limitations associated with the pandemic, we also discuss the opportunities created by this particular situation, in the context of reforms called for in forensic psychiatry in Poland (9).

## Forensic Psychiatric Care in Poland

Poland's forensic psychiatric inpatient care (2) is a three-step system, composed of maximum (high), enhanced (medium), and basic (low) security units, caring for 2,929 inpatients. Placement in a psychiatric institution is possible only when it becomes necessary to prevent reoffending which leads to high social harm, where other legal measures are not sufficient to achieve this goal. The legal framework allows detention (involuntary psychiatric forensic treatment) for clinically unwell offenders at the time of the criminal act or for those with diminished criminal responsibility due to a mental disorder in cases of severe crimes. For mentally unwell offenders [defined as: “at the time of committing a prohibited act, an offender was incapable of recognizing its (the act's) significance or controlling their conduct because of a mental disease, mental deficiency or another mental disturbance”], if the forensic psychiatric evaluation concludes that the risk of reoffending is high, it is possible to apply preventive measures which include detention and treatment in some of the 50 forensic institutions in Poland. Psychosis is the most prevalent diagnosis, followed by intellectual disability, dementia or other serious mental disorders. Outpatient forensic treatment is also possible – in both outpatient and in-patient settings the duration of the preventive measure is not predetermined. The Board for Preventive Measures, an institution under the direct control of the Ministry of Health, provides a central mechanism for the allocation of inpatients, determining the level of security for patients starting their detention, the place for prolongation of detention and making recommendations to competent courts regarding the discharging or transferring of inmates.

## The Impact of the Pandemic on the Mental Wellbeing of the General Population – Recommended Interventions

The impact of the pandemic on the mental state of the population can be quite precisely determined, and the core areas for short and long term intervention planning are organizational, informative and medical (4). Interventions should be adapted for the following groups of people: the infected and sick hospitalized and non-hospitalized; the infected (carriers); the asymptomatic or oligosymptomatic; the families of sick people (COVID-19); individuals under collective and home quarantine; people suffering from other somatic diseases (risk group) and hospitalized for other diseases; nursing home residents; individuals over 60 years old; children and youths; arrested individuals (people deprived of personal liberty); military services (the police, army, border guards, city police); health and sanitary services staff; volunteers.

Each of the above mentioned groups has different psychological needs, a range of symptoms of mental discomfort, and different burdens related to the SARS-CoV-2 virus pandemic. There can be no doubt that a different organizational, informational, medical and, in certain cases, therapeutic message and regime must be adapted for each group. The marginalization of needs and strategic omissions in these areas today may cause, in accordance with the dynamics of psychophysiological responses to stress, increasing mental disorders starting from as early as 6 months of age, after the danger disappears, manifesting itself over the ensuing years. These long-term mental effects of trauma, requiring treatment, may affect 20 or more percent of persons forced to deal with the pandemic. Having already undertaken preparations for the development of standards and therapeutic programs necessary for the future, after the epidemic risk has been inhibited, the systems increasing the sense of security here and now should be implemented first.

## Recommendations for Risk Management of SARS-CoV-2 Coronavirus Infection for General Psychiatry

Recommendations issued by the Polish Ministry of Health answer the need to develop guidance for psychiatric care providers at risk of infection, along with recommendations based on those issued for general health care units (10, 11). As stated by the authors of this article, the document should serve as a guide for the development of internal procedures by psychiatric care providers. It has, however, been accepted that if some organizational aspects are not possible to implement, equivalent procedures are acceptable, e.g., those approved by a provincial or national consultant in the field of infectious diseases or epidemiology.

Recommendations are divided into sections, relating to hospital admission algorithms, in-patient treatment, daily stay units, and out-patient services.

## Safety Recommendations for Medical Workers in Psychiatric Establishments

It is recommended that staff in emergency departments, triage centers and general psychiatric establishments avoid direct contact with other persons wherever possible. The body temperature of staff should be checked as they enter their place of work. Local safety procedures should be clearly explained to security staff. Shift work is possible – teams working in cycles of e.g., 7 days or permanent designated teams working together. The ways in which the daily work of any given unit is organized should exclude direct contact between members of staff (during training activities, briefings, etc.) in order to avoid infections between different teams. Videoconferencing is the preferred form of hosting meetings. SARS CoV-2 genetic tests should be performed according to sanitary procedures.

## Admission to Psychiatric Departments

**Figure 1:**
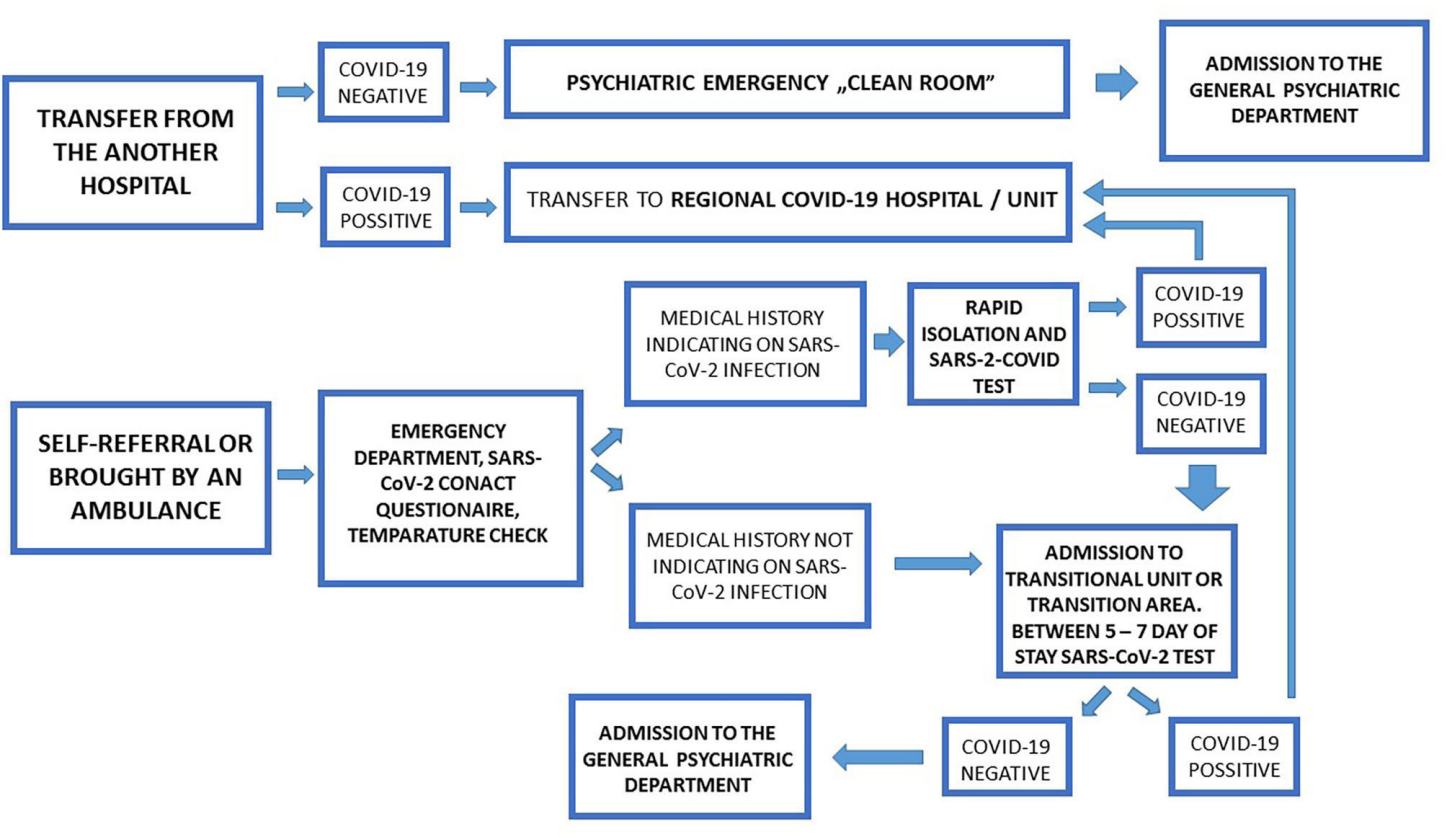
General psychiatry admission pathways.

Recommendations have been developed for two patient admission paths: (1) admission (transfer) from another hospital, (2) patients brought in by ambulance or patients who arrive at emergency departments by themselves (Figure 1).

In the first case, patient transfers between hospitals should take place after telephone consultation to confirm admission to a psychiatric hospital. Hospitals/psychiatric wards should have a designated medical doctor to call other therapeutic entities around the clock (this may be a doctor on duty). If a patient is transported from another hospital, he or she should have had a SARS-CoV-2 test performed at that hospital at least 2 days before being transported. If the result for SARS-CoV-2 is negative, the patient should be admitted to the hospital/psychiatric ward by a so-called “clean room.” If the result for SARS-CoV-2 is positive, the patient should be taken to a hospital or psychiatric ward designated by a governor as the Regional COVID-19 Hospital. Should a patient be brought in by the emergency medical team, or when the patient has independently reported to the emergency room of a psychiatric hospital, the emergency medical staff will conduct an interview on the hospital's phone/intercom, or else complete a questionnaire and measure the person's temperature in a non-contact manner (without touching the skin, in cases of patients with a caregiver/parent this will also apply to the caregiver/parent). An asymptomatic patient who does not meet the admission criteria should be referred to the ambulant service. Patients who are asymptomatic and need to be admitted to a hospital/psychiatric ward should be referred to a designated transitional unit or transition area after admission. In this unit/transit area the following staff requirements apply: a mask, apron and gloves. Patients who are asymptomatic and require admission to hospital/psychiatric ward, but have been in contact with persons with a confirmed SARS-CoV-2 infection, following a medical examination should be tested for SARS-CoV-2 and kept in isolation within the emergency room. Patients requiring admission to a hospital/psychiatric ward, who meet the “COVID-19 suspect case criteria” after a medical examination, should be tested for SARS-CoV-2 and isolated within the emergency room. Isolated patients with a confirmed SARS-CoV-2 infection, or if they meet the criteria of a “probable case,” should be transported to a Regional COVID-19 Hospital. When isolated patients receive a negative result, they are to be transferred to the transition unit or the transition area of the unit. Between the 5th and 7th day of hospitalization in the transition unit or the transition area of the unit patients should be tested for SARS-CoV-2—if they receive a negative result, they should be transferred to a target unit—if they receive a positive result, they should be transported to a Regional COVID-19 Hospital or COVID-19 department designated by a governor.

### Ambulant Psychiatric Service

Recommendations impose the use of telemedicine on out-patient services, but when it's necessary, on-site visits are possible with special safety precautions. Before each appointment, a COVID-19 interview should be conducted; appointments should be made for specific hours, reducing the number of patients in waiting rooms; the body temperature of people using the clinic (including staff – before starting work) should be measured; family members should not enter the outpatient clinic (except for those attending with children and adolescents and adults with special needs), only one person is allowed to attend outpatient clinics with a child; patients/other persons should use masks/other coverings of the nose and mouth, if a patient comes in without a mask, a mask should be given to the patient; personnel should use appropriate personal protective equipment; surfaces should be disinfected and rooms must also be ventilated; a distance of two meters is to be maintained between people in the clinic; unnecessary objects which may be conducive to the transmission of infection (for example, toys, newspapers, leaflets, etc.) should be removed from waiting rooms.

## Day-care in Psychiatric Centers

According to recommendations, a remote interview (using telephone or intercom) should be conducted every day before entering the ward for each patient. It is necessary to measure the temperature in a non-contact manner for all patients and caregivers. It is recommended that each patient come to the daily-care center wearing a protective mask, hands washed with disinfectant – during therapeutic activities, a minimum space of 4 m^2^ should be provided for each patient. Personnel should use appropriate protective equipment – in accordance with the relevant part of The Agency for Health Technology Assessment and Tariff System (AOTMiT, following the model of U.S. Food and Drug Administration) guidelines and recommendations (protective mask and/or visor). Surfaces should be disinfected systematically and rooms must also be frequently ventilated, just as in ambulant services. The conditions governing requirements for the work done by day-care centers, like the number of hours and the nature of the activities, should be flexible, in accordance with general requirements, depending on the recommendations made by management teams and professional center staff. Lunch can be swapped for a meal package; medication for the whole week may be given to the patient once a week; patients with confirmed SARS-CoV-2 infection who meet the criteria of a suspect or probable case cannot be admitted to day-care units.

### Specific Recommendations for Forensic Psychiatric Units

**Figure 2:**
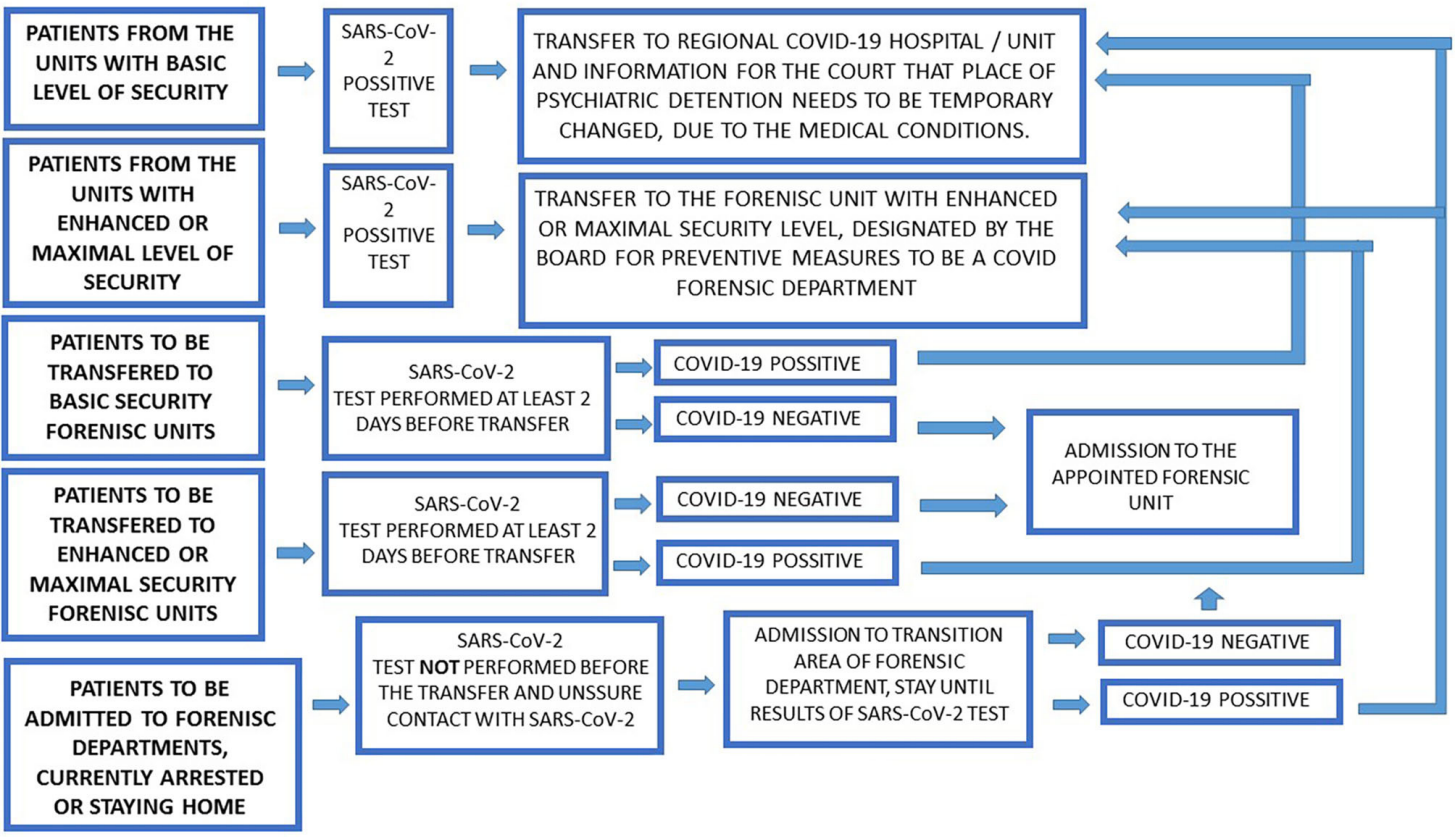
Forensic psychiatry admission and transfer pathways.

In addition to the general recommendations for psychiatric hospitals, in the case of forensic psychiatric units the transfers of patients between forensic institutions and security levels should be restricted to cases which are beyond any doubt. It is recommended that periodic opinions issued for courts (to be issued every 6 months) should include the risk of epidemics. Patients staying in wards with a basic level of security who test positive for SARS-CoV-2 should be transferred to a hospital or psychiatric ward designated by the governor as the Regional COVID-19 Hospital. At the same time, competent courts (penitentiary judges) should be informed of the necessity to change the place of detention where protective measures are taken until the symptoms of COVID-19 disappear. Transfer between forensic psychiatric institutions and from custody and prison to forensic institution is possible only for patients who have been tested for SARS-CoV-2 genetic tests in the referring institution at least 2 days before transfer. Patients with a positive SARS-CoV-2 test and transfer orders to units with a basic level of security should be transferred to a hospital or psychiatric ward designated by a Governor as the Regional COVID-19 Hospital instead. Patients with orders for transfer to units with enhanced or maximal security level, in cases of positive SARS-CoV-2 tests, should be redirected to units designated by The Board for Preventive Measures, an institution under the direct supervision of the Ministry of Health, and if there are no vacancies in designated units, patients should wait until transfer at their current place of stay. Patients being admitted to psychiatric forensic units who have not been tested for SARS-CoV-2 and whose medical history is uncertain or who are under quarantine, and are being held in a police detention center or place of residence, should be treated as suspected COVID-19 cases and admitted to the appropriate triage zone until a SARS-CoV-2 test is obtained. In enhanced and maximum security level psychiatric institutions the recommendation is for a 1-bed triage zone with a separate room for dedicated staff and separate sanitary facilities to be set up. This zone is designated for patients awaiting the results of tests for SARS-CoV-2, and they should stay in this triage zone before joining the common areas of forensic departments. In addition, it is recommended transition subunits be organized for patients who have recently been admitted or who display symptoms of infection during ongoing forensic treatment. It is recommended that SARS-CoV-2 tests be performed every 5–7 days. In case of symptoms of infection, it is recommended the test be performed earlier. It is recommended that such a subunit, located in the structure of the hospital, should have a dedicated nursing and care team. During an epidemic, it is recommended meals be provided to patient rooms in order to minimize contact with other patients and staff. Forensic departments, due to their high occupancy and high potential epidemic risk, require procedures which may violate patient rights. In such exceptional conditions, it is recommended that visits be suspended, along with temporary passes (temporary leave) from hospitals – replaced with 24 h quarantine packages to provide psycho-educational activities for patients at risk of infection. Transfer pathways are presented in Figure 2.

## Discussion

The development of new guidelines and their implementation has become a necessity in times of pandemic. As forensic psychiatric patients are a particularly traumatized social group—suffering from psychotic disorders, personality disorders, intellectual disabilities (12–14) both in Poland and other European countries—actions taken to reduce the risk of SARS-CoV-19 infection seem to be a necessity, also in the context of distant effects of the pandemic, including trauma and escalation of mental disorders (15, 16). The length of stay for forensic inpatients is affected by clinical factors, as well as by external factors such as juridical systems, criteria of admission, length of court proceedings, resources of general psychiatric care staff and social and community support (17). Restrictions in personal liberty resulting from involuntary psychiatric forensic treatment has to be carried out with full respect for human rights and cannot exceed the period which is absolutely necessary – the SARS-CoV-2 pandemic state should not have a significant influence on length of stay, or at least this impact should be minimized. To achieve this, it is essential both to introduce appropriate recommendations and guidelines and to closely monitor the situation in the correctional facilities, as exemplified by actions taken by National Commission on Correctional Healthcare in the US (18–20).

Safety regulations also affect healthcare workers. Particular attention should be paid to the safety of health and sanitary services workers, as in the forensic psychiatric environment this group could potentially be a “gateway to infection,” as they usually work in more than one healthcare institution and have contact with many potentially infected individuals. In a forensic psychiatric setting, there is a relatively low risk of spreading infections by newly admitted patients, which are usually transfers from other forensic centers or jails, compared to general hospital departments admitting patients in acute states. This risk is additionally reduced by performing SARS-CoV-2 genetic tests prior to transfer and isolating admitted patients who could not be tested. Key concerns relate to potential cases where a significant number of patients are infected by staff and de-facto cannot be transferred to a hospital dedicated to COVID because of their mental condition, seeing as they pose a risk to their surroundings. The safety of personnel ensures the safety of patients. It is essential establishments provide a sufficient amount of protective equipment such as masks, gloves and aprons for personnel to minimize risk of infection. The Polish recommendations for psychiatric care, contrary to some of the recommendations in other countries (18), do not provide detailed suggestions regarding personal protective equipment to be used by staff. This aspects are regulated by other recommendations of Polish Ministry of Health and The Agency for Health Technology Assessment and Tariff System (10, 11).

This is further aided by the frequent washing and disinfection of hands, disinfection of ward surfaces, temperature measurements among personnel, all of which are clearly recommended in the guidelines. The idea of frequent screening of staff members remains open to question. In our opinion, decisions on whether to screen medical staff should be made by hospital management, based on local risk assessments. Moreover, in this case, shift work should be carried out to avoid quarantining entire teams. We are however of the opinion that shift work is possible only in short-time durations in forensic psychiatric settings. Forensic patients require constant psychological support, work on insight into criminal behaviors, improving awareness of their own psychological and mental state, group activities including occupational therapy, cognitive function training and psychotherapy. It is not possible to provide care and support necessary for the treatment process while struggling with staff shortages. The process of treating mentally disturbed offenders and returning them to freedom cannot be delayed in time and postponed until after the pandemic. In order to minimize the risk of infection transmission by forensic psychiatric personnel from other hospital units where staff often work extra hours, it is worth considering financial incentives (additional remuneration) for staff who decide to give up other jobs during the pandemic. To a large extent, this could help overcome staff shortages. Unfortunately, the use of financial incentives was not included in the recommendations. Although this would certainly be valuable, the recommendations make little reference to the individual and group therapy for forensic inpatients. For the safety reason, the tele-medicine approach, as the contact with psychologists and other therapists using telephone and online communicators, worked well in the department where the authors work on a daily basis during the pandemic. It is certainly worth introducing such solutions.

During the pandemic, an increase in psychological problems among health workers, mainly nurses, as well as increased levels of anxiety, depression, insomnia, chronic fatigue and other stress related symptoms, have been observed. Adequate psychological support for medical personnel involved in the fight against the epidemic should be ensured, regardless of resource concerns.

Another challenge that is related to the COVID epidemic is the need to ensure the safety of patient isolation during sanitation procedures, transport, and transfer between wards. Counteracting possible patient escapes requires additional resources and sufficient staff during the pandemic. This becomes an extraordinary challenge given the numerous staff absences caused by the disease. However, this must not be forgotten.

These recommendations were preceded by a discussion on both the medical aspects related to the risk of virus spread and the ethical aspects of limiting patients' rights. Poland's Human Rights Ombudsman Office pointed out the need to provide individual protective equipment for staff of forensic units and the need to inform patients about the risk of COVID-19. In addition, the office asked, among other things, whether: tests for coronavirus are being carried out among forensic inpatients; quarantined persons are included in the group of forensic inpatients; transfers of patients between forensic centers are still taking place or whether patients attend court meetings. The ethical and legal consequences of introduction of pandemic-related restrictions directly affecting patients' personal freedom was widely discussed. The suspension of visits and passes, isolation of patients in the transitional units, the quarantine of shipments and restriction of group activities is a consequence of a state defined in Polish law as one of “greater need” which assumes it may be necessary to sacrifice one “good” protected by law for the sake of another, on the condition that the “good” which is in this way protected does have unarguably greater value. The need to continue functioning under pandemic conditions may force positive changes in the system of forensic psychiatry, as postulated by Kennedy et al. (7). The introduction of ICT solutions, such as telepresence in courts, can significantly simplify certain procedures and reduce the operating costs of the forensic care system. However, it should be remembered that it is not always possible to effectively replace direct contact with a therapist through ICT solutions. And even in isolation, medical and therapeutic care must be provided to all possible extent while ensuring safety procedures are followed. In other cases, forensic psychiatric care will lose its medical aspect and be reduced to the process of isolating offenders.

The guidelines discussed above, although restrictive in terms of temporary restrictions on patient rights and probably not easy to implement in clinical conditions, may become an important element in the fight against pandemics. In terms of recommendations for forensic psychiatric care, the guidelines place emphasis both on safety within the institution and on protecting staff and patients from transmission of infections from outside, as is the case with psychiatry.

If the severity of the pandemic decreases, considering the specifics of forensic psychiatric wards, emphasis should possibly be shifted toward protecting establishments and staff from external sources of infection, while at the same time reducing restrictions inside the wards.

## Author Contributions

JH: co-elaboration of guidelines, concept of manuscript, correction of the manuscript, and acceptance of final version. PG: collection of resources, literature search, and preparation of the text. All authors contributed to the article and approved the submitted version.

## Conflict of Interest

The authors declare that the research was conducted in the absence of any commercial or financial relationships that could be construed as a potential conflict of interest.
